# Clinical Course of Opportunistic Infections—Toxoplasmosis and Cytomegalovirus Infection in HIV-Infected Patients in Slovakia

**DOI:** 10.3390/pathogens8040219

**Published:** 2019-11-04

**Authors:** Katarína Šimeková, Elena Nováková, Róbert Rosoľanka, Jana Masná, Daniela Antolová

**Affiliations:** 1 Clinic of Infectology and Travel Medicine, Comenius University Bratislava, Jessenius Faculty of Medicine and University Hospital in Martin, Kollárova 2, 036 01 Martin, Slovakia; Katarina.Simekova@jfmed.uniba.sk (K.Š.); Robert.Rosolanka@jfmed.uniba.sk (R.R.); jankamasna@gmail.com (J.M.); 2 Institute of Microbiology and Immunology, Comenius University Bratislava, Jessenius Faculty of Medicine and University Hospital in Martin, Kollárova 2, 036 01 Martin, Slovakia; Elena.novakova@jfmed.uniba.sk; 3 Institute of Parasitology of Slovak Academy of Sciences, Hlinkova 3, 040 01 Košice, Slovakia

**Keywords:** HIV/AIDS, persistent generalized lymphadenopathy, toxoplasmosis, Cytomegalovirus, immunodeficiency

## Abstract

The HIV/acquired immunodeficiency syndrome (AIDS) pandemic has affected the health status of the population in many countries. Early symptomatic HIV infection includes persistent generalized lymphadenopathy (PGL), which can be associated with opportunistic infections, e.g., toxoplasmosis and Cytomegalovirus (CMV) infection. This study followed the occurrence of PGL, toxoplasmosis, and Cytomegalovirus infection in 32 HIV-positive patients and analyzed the clinical signs of disease in relation to the number of CD4 T lymphocytes. In monitored patients, the average number of CD4 T lymphocytes was 940.8 ± 396.7/µL of blood. Severe immunodeficiency was recorded in four persons, who also suffered from colitis and/or retinitis and pneumonitis. The number of CD4 T cells in patients with PGL was significantly lower than that in patients without lymphadenopathy. In 6 (18.8%) IgM and 11 (34.4%) IgG *Toxoplasma gondii* seropositive patients, the number of CD4 T lymphocytes was significantly lower than that in seronegative patients. The presence of IgM and IgG antibodies to Cytomegalovirus was recorded in all examined patients, and CMV infection clinically manifested in five persons. The occurrence of PGL, the higher viral load, and seropositivity to *T. gondii* were significantly related to decline in the CD4 T lymphocyte number. The clinical course of the diseases was influenced by the status of the patient’s immunodeficiency and suggests ongoing immunosuppression and possible reactivation of both infections in all patients.

## 1. Introduction

The HIV/AIDS pandemic has severely affected health development and eroded improvements in life expectancy, particularly in countries with the highest prevalence of infection. Human immunodeficiency virus (HIV) infection encompasses an acute phase that lasts for months, followed by a clinically latent phase that typically lasts for a few years and, ultimately, by the collapse of immune system that characterizes acquired immunodeficiency syndrome (AIDS) [1]. Early symptomatic HIV infection includes persistent generalized lymphadenopathy (PGL), often the earliest symptom of primary HIV infection. PGL is defined as lymph node enlargement involving two or more non-contiguous sites, with at least 3 months’ duration [2]. The differential diagnosis of PGL in the HIV-infected population typically falls into one of three major categories: infection, malignancy, or reactive changes [3].

Different opportunistic infections such as tuberculosis, toxoplasmosis, disseminated fungal infections, atypical mycobacterial infections, Cytomegalovirus infection, etc., as well as immune reconstitution inflammatory syndrome (IRIS), may be associated with lymphadenopathy [4,5].

*Toxoplasma gondii* is an obligate intracellular protozoan parasite that infects all warm-blooded animals and has worldwide occurrence. The parasite infection is common in humans, but the majority of cases in immunocompetent persons are asymptomatic, or various mild symptoms may be observed. The illness course is usually mild, with “flu-like” symptoms that last for weeks to months. Sometimes also localized or generalized lymphadenopathy occurs; swollen lymph nodes are commonly found in the cervical, occipital, or axillar region. The ocular form usually presents as chorioretinitis, complicated vitreitis, or hemorrhage appearing [6,7]. Immunocompromised persons may experience severe symptoms if they are infected with *Toxoplasma*. Persons who acquire HIV infection and were not previously infected are more likely to develop a severe primary infection, while persons who were infected before they become immunosuppressed are at risk of developing a relapse (reactivation) of toxoplasmosis [8]. Reactivation of chronic *Toxoplasma* infection in HIV-infected persons is most often connected with encephalitis with focal neurological abnormalities, sometimes with fever, defects of the visual field, and defects of cerebellum function, with neuropsychiatric symptoms. The extracerebral form can also occur, mostly presented as chorioretinitis with multifocal and bilateral lesions of the optic nerve. Sometimes pulmonary symptoms or even polymyositis and hepatitis may occur [9,10].

Cytomegalovirus (CMV) belongs to the Herpesviridae family and occurs throughout all geographic locations and socioeconomic groups. The virus infects between 60% and 70% of adults in industrialized countries and up to 100% in emerging countries [11]. The course of the infection is usually without clinical signs in healthy people but can be life-threatening for the immunocompromised, such as HIV-infected persons, organ transplant recipients, or newborn infants [12]. After the primary infection, CMV establishes a lifelong latent infection with possible reactivation and reinfection. The acute and latent phases of infection in immunocompetent individuals are usually asymptomatic; however, there are reports that infection can be associated with hepatitis, neurological and intestinal symptoms, immunosenescence, functional impairment, etc. [13,14,15]. Reactivation of CMV may occur at any time during life, although the risk is higher in the setting of systemic immunosuppression, either iatrogenic or secondary, such as with AIDS [16]. It is usually accompanied by asymptomatic excretion of the virus in the urine, saliva, and other body secretions. Later, when the number of CD4 T lymphocytes decreases below 50–100/µL of blood, impairment of the organs appears. Cytomegalovirus retinitis causes blurred vision; CMV esophagitis manifests as painful swallowing, and CMV colitis or enterocolitis, which affects approximately 5–10% of AIDS patients, is connected with pain and diarrhea. Impairment of the central nervous system (CNS) is exhibited as polyradiculitis, myelitis, or ventriculoencephalitis [17].

The aim of the work was to monitor the occurrence of persistent generalized lymphadenopathy, toxoplasmosis, and Cytomegalovirus infection in HIV-positive patients and analyze the clinical signs of the disease in relation to the number of CD4 T lymphocytes.

## 2. Results

The results of serological and clinical examinations of 32 patients (30 men and 2 women) monitored at the Outpatient Department for Monitoring and Treatment of HIV-Infected Persons at University Hospital in Martin, Slovakia, are reported in Table A1 of the Appendix A. All patients were on antiretroviral therapy (ART) for at least half a year. The longest duration of therapy was 8 years. Ten patients were receiving first-line therapy, and 12 and 10 persons were on second- and third-line therapy, respectively. No correlation between ART therapy regimen and the occurrence of opportunistic infections was observed.

In the group of all observed patients, the average number of CD4 T lymphocytes was 940.8 ± 396.7 per µL of blood, ranging from 64/µL to 1440/µL. Severe immunodeficiency (a decline in CD4 T lymphocytes below 200/µL of blood) was recorded in three persons, and in one patient the number was close to this level (216/µL of blood). All four of these patients suffered from colitis, which was also confirmed by histological examination. Colitis was also documented in patient No. 9, whose CD4 lymphocyte count reached only 252 per µL of blood. Patient No. 26, with 118 CD4 T lymphocytes/µL of blood, suffered from retinitis with severely blurred vision, and CMV pneumonitis was diagnosed in patient No. 23, who had a count of 64 CD4 T lymphocytes.

Persistent generalized lymphadenopathy was recorded in 22 patients. Statistical analysis of the CD4 T lymphocyte counts showed a significantly lower number (*p* = 0.0005) of CD4 T cells in patients with PGL than in patients without lymphadenopathy. The mean number of CD4 T lymphocytes in people with PGL was 496.6 ± 229.3 per µL of blood and ranged between 64 and 942. In patients without signs of lymphadenopathy, the mean number of CD4 T cells was higher (958.1 ± 430.5/µL of blood), ranging from 184 to 1440 per µL of blood.

The number of CD4 T lymphocytes also showed a statistical relationship with the antiretroviral therapy regimen (*p* = 0.028). The mean number of CD4 T cells in patients on first-line ART was 722.3 ± 354.0/µL of blood, while in those receiving second-line therapy it was 780.8 ± 375.7/µL, and in patients on third-line ART, it was only 391.4 ± 386.5 CD4 per µL of blood.

Viral load over the detection limit of the test (40 copies/mL) was detected in nine patients, ranging between 1200 and 320,000 copies (57,911 on average). Five patients were ART-treated for only 0.5–1 year, while in four patients the treatment had lasted 3, 5, or 8 years. The number of CD4 T lymphocytes in patients with an undetectable viral load (736.1 ± 369.8/µL of blood) was significantly higher (*p* = 0.017) than in those with a higher number of viral copies in the blood (397.3 ± 246.3/µL of blood). All persons with a detectable viral load suffered from PGL or striking PGL, and in four of them colitis also was recognized.

An increased level of IgM antibodies to *Toxoplasma gondii*, signalizing the acute form of the infection, was detected in six (18.8%) persons, and in one patient the level of antibodies was within the gray zone. Striking generalized lymphadenopathy was reported in all seven of these patients. Positivity to IgG antibodies to *T. gondii* was detected in 11 (34.4%) patients, and in three others the level of antibodies was within the gray zone. Four patients (12.5%) were positive to both IgG and IgM antibodies. A significant difference (*p* = 0.0074) in the CD4 T lymphocyte count was revealed between patients seropositive to *T. gondii* (IgM and/or IgG positive) and those who did not produce antibodies to *Toxoplasma*. Patients positive to *Toxoplasma* had on average 488.3 ± 274.0 CD4 T lymphocytes per μL of blood (range 64–942), while seronegative persons had 826.1 ± 387.4 CD4 T cells/μL, ranging between 328 and 1440. The mean number of CD4 T lymphocytes in the seven patients with IgM anti-*Toxoplasma* antibodies and striking lymphadenopathy was 418.3 ± 336.4 cells/μL. In seven of nine patients with a detectable viral load, the presence of IgG and/or IgM antibodies to *T. gondii* was recorded, but there was no statistical relationship between the viral load and seropositivity to *Toxoplasma* (*p* > 0.05).

In all the examined patients (100%), positivity to IgM and IgG antibodies to Cytomegalovirus was recorded.

## 3. Discussion

The first cases of AIDS were reported in 1981, and since that time, infection with HIV has grown to pandemic proportions. Globally, approximately 36.9 million (31.1–43.9 million) people were infected with HIV at the end of 2017 [1]. In Slovakia, the monitoring of HIV and AIDS began in 1985. At the end of 2018, a total of 1072 people were suffering from the infection. Of that number, 904 patients were of Slovak nationality and the rest were foreigners. In 118 cases, the HIV infection developed into AIDS [18].

After infection with HIV, it takes 4–6 months until a steady state of viraemia is reached in a patient. Then, the plasma virus load level usually remains stable for several years, and the infected person is asymptomatic during this period. Although plasma virus load levels do not rise, the constant multiplication of the virus causes the destruction of CD4 T lymphocytes. Their replenishment cannot keep pace with their loss, and the result is a gradual decline of CD4 T lymphocytes in peripheral circulation. Therefore, CD4 T lymphocyte count is an important parameter of HIV disease progression. A normal range for CD4 cells is about 500–1500 per μL of blood. The gradual decrease in CD4 T lymphocytes cells (below 200 cells/μL) ultimately results in a loss of control over immune response and the development of various opportunistic infections. This is the terminal stage of HIV infection, which is called AIDS [19,20].

Lymphadenopathy is one of the most common manifestations of HIV/AIDS with different underlying pathogeneses. Besides tuberculosis, malignant lymphoma, reactive hyperplasia, Kaposi sarcoma, and IRIS, opportunistic infections are among the causes resulting in lymphadenopathy [5,21]. Persistent generalized lymphadenopathy was recorded in 22 of the patients monitored in the present study. Analysis of the number of CD4 T lymphocytes showed a significantly lower number of CD4 cells in patients with PGL than in those who were not suffering from lymphadenopathy. Generally, the stage of HIV infection can be divided according to the CD4 T lymphocyte count. The number of CD4 cells in the early stage is >500/μL; in an intermediate stage it varies between 200 and 500/μL; and in an advanced stage it is below <200/μL [22,23]. The mean number of CD4 T cells in our patients with PGL was 496.6 (64–942), meaning that in the majority of them the intermediate stage of the disease was in progress.

In the study, the number of CD4 T lymphocytes correlated with the viral load, as a higher number of CD4 cells was detected in patients with undetectable viral load (736.1/µL of blood) than in those with a higher number of viral copies in the blood (397.3/µL of blood). The differences between CD4 T lymphocyte number and the ART regimen were also recorded; a higher number of CD4 was documented in persons receiving second-line ART than in those on first- and third-line therapy.

As mentioned above, persistent generalized lymphadenopathy is often connected with different opportunistic infections, with toxoplasmosis and Cytomegalovirus infection being considered frequent causes of lymph node enlargement [4]. In the presented study, IgG anti-*Toxoplasma* antibodies were detected in 11 (34.4%) monitored patients. Increased levels of IgM antibodies, signalizing the acute form of *Toxoplasma* infection, were detected in six (18.8%) persons, and in one patient the level of antibodies was within the gray zone (dubious result). Four people were positive to both IgG and IgM antibodies, and in three of them a severe drop in the CD4 T lymphocyte count (<200/µL of blood) manifested. In all patients with a positive or dubious level of IgM antibodies, striking generalized lymphadenopathy was documented. The occurrence of both IgM and IgG antibodies together with the severe drop in CD4 T cells and the presence of striking generalized lymphadenopathy in four of presented patients indicated the reactivation (relapse) of toxoplasmosis. Moreover, in patients with *T. gondii* infection (IgM and/or IgG positive), a significantly lower number of CD4 T lymphocytes was detected (488.3/µL of blood) than in seronegative patients (826.1/µL of blood), and the drop in cell number was much more obvious in patients with IgM seropositivity (418.3/μL of blood).

In general, the clinical course of toxoplasmosis in HIV-positive patients significantly depends on the CD4 T lymphocyte count. A decline below 100 cells per μL of blood causes the reactivation of latent infection, most often encephalitis. It is usually connected with the presence of different symptoms, such as nausea, headache, seizures, problems with speech, central paresis, cerebellar signs, etc. The following progress of symptoms depends on the progression of immunodeficiency and the efficiency of treatment [10,17]. Although in our patients no symptoms of encephalitis appeared, the occurrence of striking PGL in patients with IgM antibodies indicated the onset of reactivation of the disease. Moreover, our results also clearly suggest a relationship between the number of CD4 T lymphocytes and toxoplasmosis reactivation.

Systemic immunosuppression in HIV-positive patients can also cause the reactivation of the Cytomegalovirus at any time during the life of an infected human host. In the analyzed group, 100% positivity to both IgM and IgG antibodies to CMV was documented. Such high seropositivity to IgG suggests repeated contact of all monitored patients with an infective agent, while the presence of IgM antibodies indicates reactivation of the infection. Clinical manifestation, namely CMV colitis, CMV pneumonitis, and CMV retinitis, was diagnosed in five patients. In two of them, the concurrent occurrence of colitis and pneumonitis or retinitis was reported. While colitis alone manifested in patients with numbers of CD4 T lymphocytes ranging between 184 and 252 per µL of blood, a more significant decline (to 118 or 64 CD4/µL) resulted in the occurrence of another complication, retinitis or pneumonitis. The reactivation of the disease accompanied by the production of IgM antibodies, as well as the occurrence of clinical signs of CMV infection in the analyzed group, also confirmed the assumption of ongoing immunosuppression in all our patients.

## 4. Material and Methods

The analyzed group comprised all patients monitored in 2018 at the specialized Outpatient Department for Monitoring and Treatment of HIV-Infected Persons at University Hospital in Martin, Slovakia. A total of 32 patients were involved in the study. All patients agreed with the survey and signed the informed consent; no identifying data are presented in the paper. The data and results of the examinations were collected during the regular control visits of patients in 2018. The analyzed group was composed of 30 men and 2 women. Their age varied between 20 and 56 years, and the average age was 32 years. The study focused on analyses of the number of CD4 lymphocytes, the presence of persistent generalized lymphadenopathy, viral load, and the levels of IgM and IgG antibodies to *Toxoplasma gondii* and Cytomegalovirus.

The study was in accordance with the 1975 Declaration of Helsinki, as revised in 2013, and was approved by the Ethics Committee of Comenius University Bratislava, Jessenius Faculty of Medicine in Martin (No. EK 4/2019).

The number of CD4 T lymphocytes in blood samples was analyzed using the standard flow cytometry method and is stated in absolute number of cells per microliter of blood.

Viral load in the blood samples was assessed using Xpert^®^ HIV 1 Viral load (Cepheid, Sunnyvale, CA, USA) according to the manufacturer’s instructions. The detection limit of the test is 40 copies in 1 mL of plasma.

The levels of IgM and IgG anti-*Toxoplasma gondii* antibodies were determined using the Architect Toxo IgG and Architect Toxo IgM (Abbott, IL, USA) diagnostic kits. According to the producer’s instructions, the results of the test for IgM antibodies were calculated using the Architect i System and reported as sample to cut-off (S/CO). Specimens with results of ≥1.00 S/CO were considered reactive for IgM antibodies to *Toxoplasma gondii*, and specimens with results in the interval 0.83 ≤ *x* < 1.00 S/CO were considered to be in the gray zone. For IgG antibodies, the results were calculated in International Units (IU). Specimens with concentration values of ≥3.0 IU/mL were considered reactive for IgG antibodies to *Toxoplasma gondii*, which indicates past or acute infection. Samples with concentration values from 1.6 to 3.0 IU/mL were considered to be in the gray zone and may contain low levels of IgG.

Antibodies to CMV were tested using the Architect CMV IgG and Architect CMV IgM (Abbott, IL, USA) reagent kits. According to the manufacturer’s instructions, the default result unit for the Architect CMV IgM assay is Index. Specimens with concentration values of ≥1.00 Index were considered to be reactive for IgM antibodies to CMV, indicating acute infection. Such individuals are potentially at risk of transmitting CMV infection. For the CMV IgG assay, the default result unit is AU/mL. Samples with concentration values of ≥6.0 AU were considered to be reactive for IgG antibodies to CMV, indicating past or acute infection.

Statistical differences between the numbers of CD4 T lymphocytes in patients were assessed using the two-tailed *t*-test and one-way ANOVA (a value of *p* < 0.05 was considered significant), which allowed for the comparison of continuous variables in two groups. The analyses were performed using the Statistica 6.0 (Stat Soft, Tulsa, OK, USA) statistical package.

## 5. Conclusions

We can conclude that the occurrence of PGL and seropositivity to *T. gondii* was significantly related to a decline in the CD4 T lymphocyte number. The clinical course of diseases was influenced by the status of the patient’s immunodeficiency; thus, the occurrence of IgM antibodies to CMV and *T. gondii* and clinical presentation of CMV and/or *Toxoplasma* infection suggest ongoing immunosuppression in all our patients and possible reactivation of both infections. Besides antiretroviral treatment for HIV infection, the clinical symptoms of CMV or *Toxoplasma* infection must be addressed by supplementation with efficient virostatic and parasitostatic treatment.

## Appendix A

**Table A1:** Results of blood analyses and occurrence of clinical symptoms in 32 HIV-positive patients.

No.	CD4 T-lymphocytes/μL of Blood	Viral Load (Copies/mL)	*Toxoplasma gondii*		Cytomegalovirus		Clinical Symptoms	Line of ART	Duration of Therapy (Years)
			IgM (S/CO) (POS > 1.00 S/CO) (GZ 0.83–1.00 S/CO)	IgG (IU/mL) (POS > 3.00 IU) (GZ 1.16–3.00 IU)	IgM (Index) (POS > 1.00)	IgG (AU/mL) (POS > 6.0 AU/mL)			
1.	672	86,000	0.14	5.4	1.450	>250	PGL	1	0.5
2.	542	BDL	0.44	0.8	2.850	>250	PGL	2	3
3.	380	320,000	0.06	0.0	1.930	>250	PGL	1	0.5
4.	1250	BDL	0.07	0.6	1.980	>250	PGL	2	4
5.	680	34,000	0.21	1.2	2.36	>250	PGL	1	0.5
6.	720	BDL	0.05	0.1	1.620	>250	PGL	2	6
7.	340	BDL	0.1	3.4	2.450	>250	PGL	2	8
8.	480	BDL	0.07	6.8	1.480	>250	PGL	2	5
9.	252	10,200	0.21	17.5	4.750	>250	Colitis with positive histology finding	3	3
10.	942	BDL	0.14	0.6	1.250	>250		1	7
11.	184	6400	0.7	349.4	6.240	>250	Colitis with positive histology finding	3	8
12.	562	BDL	0.1	10.1	1.250	>250	PGL	2	4
13.	414	BDL	0.04	12.1	1.850	>250	PGL	3	6
14.	485	BDL	0.9	0.1	2.140	>250	*Striking* PGL	*3*	*7*
15.	319	BDL	1.6	7.3	3.150	>250	*Striking* PGL	*2*	6
16.	962	BDL	0.17	0.14	1.110	>250		1	2
17.	216	1200	1.2	134.2	4.250	>250	Colitis with positive histology finding *Striking* PGL	3	3
18.	784	BDL	2.3	0.6	1.560	>250	*Striking* PGL	*2*	*3*
19.	942	BDL	2.1	0.2	1.420	>250	*Striking* PGL	*2*	*2*
20.	1250	BDL	0.6	0.1	1.110	>250		2	5
21.	1440	BDL	0.41	0.8	1.250	>250		3	6
22.	860	BDL	0.78	1.2	2.420	>250		2	4
23.	64	3000	6.2	195	3.450	>250	Colitis with positive histology finding *Striking* PGL Pneumonitis	*3*	*5*
24.	512	BDL	0.14	0.1	1.560	>250	PGL	1	1
25.	386	6400	0.17	0.6	2.320	>250	PGL	1	1
26.	118	BDL	8.4	184	5.620	>250	Colitis with positive histology finding *Striking* PGL Retinitis with severe visual disorder	*3*	*7*
27.	742	54,000	0.64	2.1	1.420	>250	PGL	1	1
28.	328	BDL	0.8	0.6	2.360	>250	PGL	3	5
29.	1121	BDL	0.4	0.1	3.120	>250		1	1
30.	413	BDL	0.1	0.07	1.410	>250	PGL	3	6
31.	826	BDL	0.07	0.44	1.230	>250	PGL	1	4
32.	1320	BDL	0.52	0.72	1.140	>250		2	2

PGL, persistent generalized lymphadenopathy; BDL, below detectable level (<40 copies/mL of blood), ART, antiretroviral therapy; POS, positive; GZ, gray zone; S/CO, sample to cut-off; IU, International Units; Index, default result unit for the Architect Cytomegalovirus (CMV) IgM assay; AU/mL, default unit for the Architect CMV IgG assay.
